# Effect of Inulin-Type Carbohydrates on Insulin Resistance in Patients with Type 2 Diabetes and Obesity: A Systematic Review and Meta-Analysis

**DOI:** 10.1155/2019/5101423

**Published:** 2019-08-27

**Authors:** Mingyue Rao, Chenlin Gao, Ling Xu, Lan Jiang, Jianhua Zhu, Guo Chen, Betty Yuen Kwan Law, Yong Xu

**Affiliations:** ^1^State Key Laboratory of Quality Research in Chinese Medicine, Faculty of Chinese Medicine, Macau University of Science and Technology, Avenida Wai Long, Taipa, Macau, China; ^2^Department of Oncology, Affiliated Hospital of Southwest Medical University, Luzhou, Sichuan 646000, China; ^3^Department of Endocrinology, Affiliated Hospital of Southwest Medical University, Luzhou, Sichuan 646000, China

## Abstract

**Background:**

Insulin resistance (IR) is a physiological condition related to type 2 diabetes mellitus (T2DM) and obesity, which is associated with high blood insulin and glucose. Inulin-type carbohydrate (ITC) is a kind of fermentable fructan that can reduce glucose and ameliorate IR in an animal model, but the effect in clinical trials is controversial.

**Objective:**

The authors conducted a systematic literature review to evaluate the effect of ITC supplementation in ameliorating IR in T2DM and obese patients.

**Methods:**

Multiple databases were queried for studies before December 25, 2018, which involved supplementation with ITC in ameliorating IR in T2DM and obese patients. Studies that involved meta-analysis of the body mass index (BMI), fasting plasma glucose (FPG), fasting insulin (FI), HbA1c, homeostatic model assessment IR (HOMA-IR), and quantitative insulin sensitivity check index (QUICKI) of T2DM subjects were included. HOMA-IR and QUICKI were identified as the primary outcomes. A systematic review was performed to evaluate the effect of ITC on IR in obese patients.

**Results:**

The database search yielded 25 studies, which met the inclusion criteria; 11 articles were meta-analyzed, and 5 other articles on T2DM and 9 articles on simple obesity were systematically reviewed. Our results did not find ITC supplementation decrease postintervention and reduction data of BMI (*P* = 0.08). However, it can significantly decrease postintervention and reduction data of FPG, FI, HbA1c, and HOMA-IR. Heterogeneity was eliminated by subgroup analysis according to baseline BMI. There was no significant difference in the amelioration of QUICKI between the ITC and control groups. However, the difference was statistically significant and the heterogeneity was eliminated after subgroup analysis according to intakes of ITC. 14 articles for a systematic review found that the results of blood glucose, insulin, and HbA1c were controversial. Only one of the seven studies on simple obesity concluded that ITC intervention significantly ameliorated HOMA-IR, while the other six did not.

**Conclusion:**

Supplementation of ITC can ameliorate IR in T2DM, especially in obese T2DM patients, but the effects are controversial in obese patients.

## 1. Introduction

Type 2 diabetes mellitus (T2DM) is considered a multifactorial disease, promoted by both genetic and environmental factors, which is characterized by chronic hyperglycemia and insulin resistance (IR) [1, 2]. The global prevalence of diabetes is estimated by the International Diabetes Federation and indicated that there are 451 million diabetic patients worldwide in 2017, of which T2DM accounts for about 90% [3]. Diet with low fiber, high fat, and sugar has been linked to obesity [4], which is a most relevant risk factor for T2DM [5]. In patients with T2DM, IR antedates the onset of overt diabetes and may represent a predictive marker for this disease [6, 7]. Obesity in patients with T2DM will aggravate IR [8]. Therefore, body control based on dietary intervention will help to ameliorate IR and improve the efficacy of hypoglycemic medication in T2DM patients [9].

One of the dietary interventions for metabolic disease is the supplementation with inulin-type carbohydrate (ITC), which is a kind of fructan that cannot be digested and absorbed in the small intestine. ITC includes inulin, oligofructose, and fructooligosaccharides, which contain fructose monomers linked by *β* (1-2) bonds [10]. Studies found that ITC could modulate the gut microbiota in animals and humans and promote the proliferation of the beneficial lactic acid-producing Bifidobacteria and Lactobacillus species [11–13]. Gut microbiota is closely related to human health and is also a microorganism that protects the intestines from colonization by exogenous pathogens. In addition, the relationship between gut microbiota and metabolic diseases such as diabetes and obesity has been confirmed by researchers [14]. Moreover, compared with other dietary fibers, ITC exhibits more advantages in glucose tolerance and IR [15]. Preclinical studies have demonstrated that a diet containing inulin can ameliorate IR in diabetic mice [16]. Oligofructose can reduce lymphocytic infiltrate into the pancreatic islets, increase the *β*-cell proliferation rate to improve insulin sensitivity and *β*-cell function [17]. The review of clinical trials also suggests that ITC supplementation has beneficial effects on metabolic syndrome in individuals with T2DM [18].

Although some studies suggest the advantage of soluble fiber supplementation on IR amelioration in individuals [19, 20], there still exists an opposite finding on ITC in T2DM or obese subjects [21–23]. To evaluate the effect of ITC supplementation on IR in T2DM and obese patients, we conducted a systematic review and meta-analysis to choose body mass index (BMI), fasting plasma glucose (FPG), fasting insulin (FI), HbA1c, homeostatic model assessment insulin resistance (HOMA-IR), and the quantitative insulin sensitivity check index (QUICKI) as the indices. The primary outcomes were HOMA-IR and QUICKI.

## 2. Methods

### 2.1. Literature Search Strategy

This systematic review and meta-analysis was conducted in accordance with guidelines set forth by the Preferred Reporting Items for Systematic Reviews and Meta-Analyses. A literature search was performed on Medline, Embase, ScienceDirect, Web of Science, Cochrane Library, China National Knowledge Infrastructure, and ClinicalTrials.gov to obtain published or grey articles before December 25, 2018. Search terms were inulin in combination with T2DM, obesity, insulin resistance, and insulin sensitivity. The search was performed by two authors independently.

### 2.2. Inclusion Criteria and Bias Evaluation

The inclusion criteria are the following: (1) the articles described as a randomized clinical trial (RCT) including a parallel and crossover study; (2) studies involved subjects with T2DM and simple obesity (but not merger T2DM); (3) subjects in an experiment group received a dietary ITC intervention compared with the control (placebo or non-ITC supplementation); (4) the outcomes included postintervention and reduction data of BMI, FPG, FI, HbA1c, HOMA-IR, and QUICKI; and (5) the articles were written in English or Chinese. Two reviewers independently assessed the articles based on the titles and abstracts and excluded studies that addressed animal or *in vitro* experiments, lacked original data, not related to ITC and IR, or duplicated studies, case reports, study protocols, or conference abstracts. The risk of bias was assessed by using the Cochrane Collaboration tool, which included seven specific items: random sequence generation, allocation concealment, blinding of participants and personnel, blinding of outcome assessment, incomplete outcome data, selective reporting, and other biases.

### 2.3. Definition and Data Extraction

Subjects in the experimental group take ITC and were allowed to treat with hypoglycemic agents during the study course. The forms of ITC (inulin or oligofructose) were either a pure food additive in their daily diet or a mixture of commodities based on ITC. The dose of the mixture was converted into pure ITC. The control group was generally supplemented with a type of digestible carbohydrate which cannot be fermented. The following important items were extracted from each included RCT: study design, subjects, sample size, baseline BMI, ITC dose and duration, and outcomes. An effort was made to email article authors to obtain data which are not shown in the published paper. All data were independently extracted by C.L. Gao and M.Y. Rao and confirmed by L. Xu and L. Jiang. Disagreements about eligibility and the extracted items were resolved by discussion between all authors, and the corresponding author (Y. Xu) ruled on disagreements.

### 2.4. Statistical Analysis

All analyses were carried out using the Review Manager software, version 5.0 (Cochrane, Copenhagen, Denmark). The FPG and FI units in all the studies were converted to be the same, and then the data were pooled to calculate the mean difference (MD) and 95% confidence interval (CI). Review Manager generated forest plots of the pooled MDs with 95% CIs for all outcomes. Allowing for heterogeneity between the studies, the data were pooled using a random effects model to facilitate generalizability of results. Statistical heterogeneity was assessed using *Q* tests and the *I*^2^ statistic. Subgroup analysis was carried out according to the clinical characteristics of the subjects to eliminate heterogeneity.

## 3. Results

**Figure 1:**
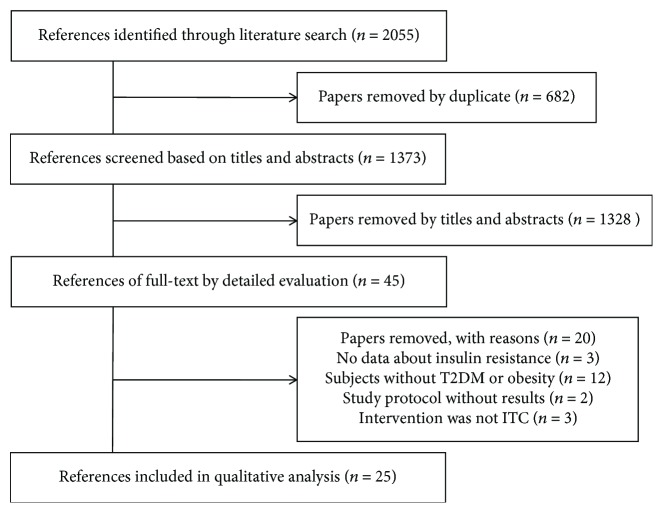
Flow diagram showing study selection.

**Figure 2:**
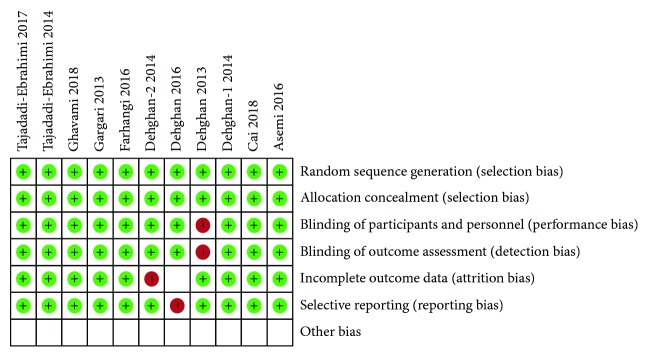
Risk of bias summary for included studies.

Our search yielded 2055 studies for an initial review. After screening titles and abstracts, 45 full-text articles were reviewed. 20 of these articles did not meet inclusion criteria, and the remaining 11 articles which comprised 634 T2DM patients were finally included in this meta-analysis [24–34] (Figure 1). In general, the included studies can be considered to have a lower risk of bias (Figure 2). Another 14 studies were systematically evaluated because the data cannot be pooled, including T2DM and obese subjects [19–23, 35–43]. The basic characteristics for all selected studies were shown in Tables 1 and 2.

### 3.1. Trial Characteristics

Eleven studies used for meta-analysis were designed to be random and double-blind or triple-blind. Only the Dehghan et al. study [26] did not report whether the study was blind. The Asemi et al. study [32] was crossover-designed, while the rest were parallel-designed. All of the studies involved patients with T2DM; six studies of which were female subjects only. The average baseline BMI of the subjects ranged from 27.69 to 31.9 kg/m^2^, and the BMI in the Ghavami et al. and Cai et al. studies [24, 29] was lower than 28 kg/m^2^. The daily dose of ITC ranged from 2.7 to 10 g, and the duration of ITC ranged from 6 to 12 weeks. The ITC dose in the Tajabadi-Ebrahimi et al. study [34] was lower than that in the other studies. Therefore, the subgroup analyses were performed based on baseline BMI and ITC daily dosage.

### 3.2. Effects of ITC Supplementation on Posttreatment BMI

**Figure 3:**
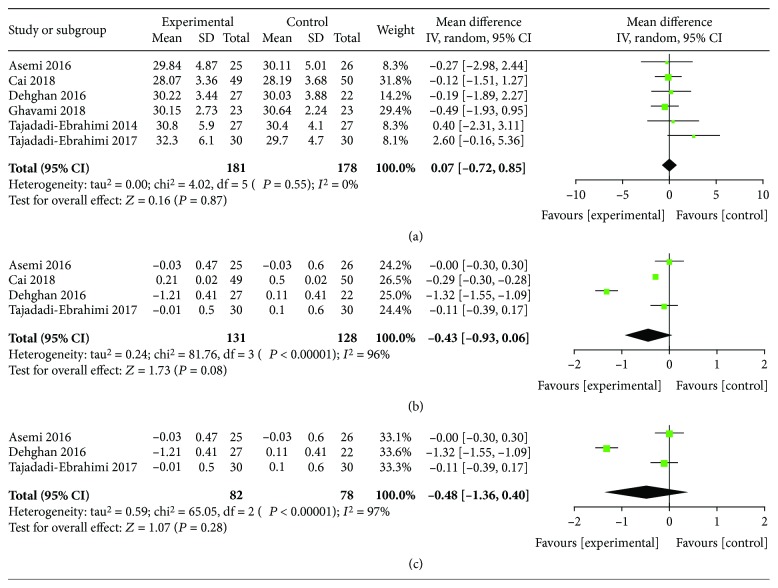
Findings of a meta-analysis of studies with continuation data on improvement in BMI for ITC vs. control groups, in terms of estimated MD and 95% CI. (a) Postintervention data of BMI, (b) reduction data of BMI, and (c) subgroup analysis for reduction data according to the baseline BMI level.

We analyzed postintervention BMI data of T2DM patients. Our results did not show that ITC supplementation decreases the BMI in whole individuals (*I*^2^ = 0%; *P* = 0.87) (Figure 3(a)). In addition, the reduction data of the BMI after ITC supplementation also showed that it was not significantly lower than that in the control group (MD, -0.43; 95% CI, -0.93-0.06; *I*^2^ = 96%; *P* = 0.08). Subgroup analysis excluding studies of the baseline BMI less than 28 kg/m^2^ also found no difference between the ITC and control groups (MD, -0.48; 95% CI, -1.36-0.40; *I*^2^ = 97%; *P* = 0.28) (Figures 3(b) and 3(c)).

### 3.3. Glucose, Insulin, and HbA1c Reduction by ITC Intervention

**Figure 4:**
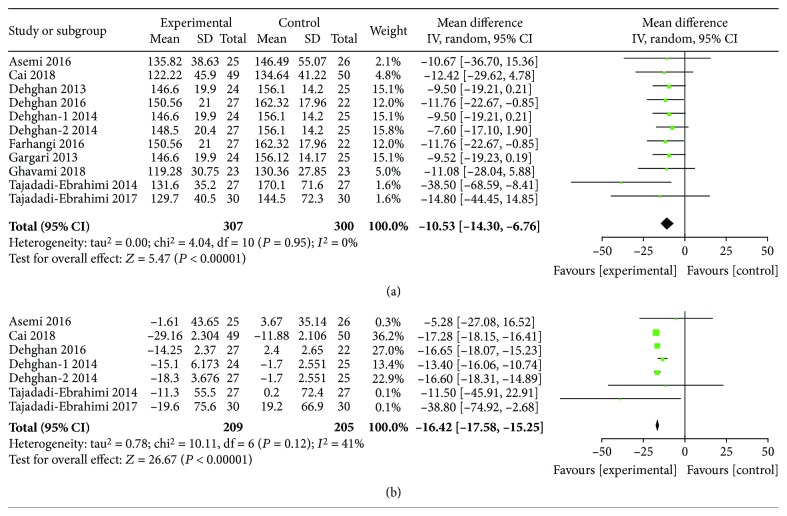
Findings of a meta-analysis of studies with continuation data on improvement in FPG for ITC vs. control groups, with estimated MD and 95% CI. (a) Postintervention data of FPG. (b) Reduction data of FPG.

**Figure 5:**
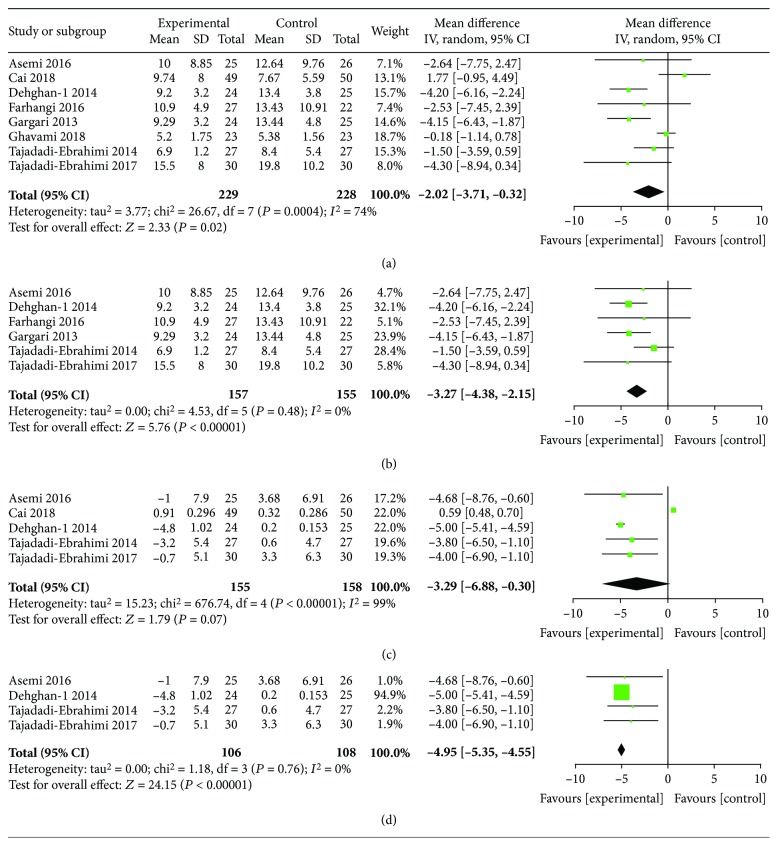
Findings of a meta-analysis of studies with continuation data on improvement in FI for ITC vs. control groups, with estimated MD and 95% CI. (a) Postintervention data of FI, (c) reduction data of FI, and (b, d) subgroup analysis according to the baseline BMI level.

**Figure 6:**
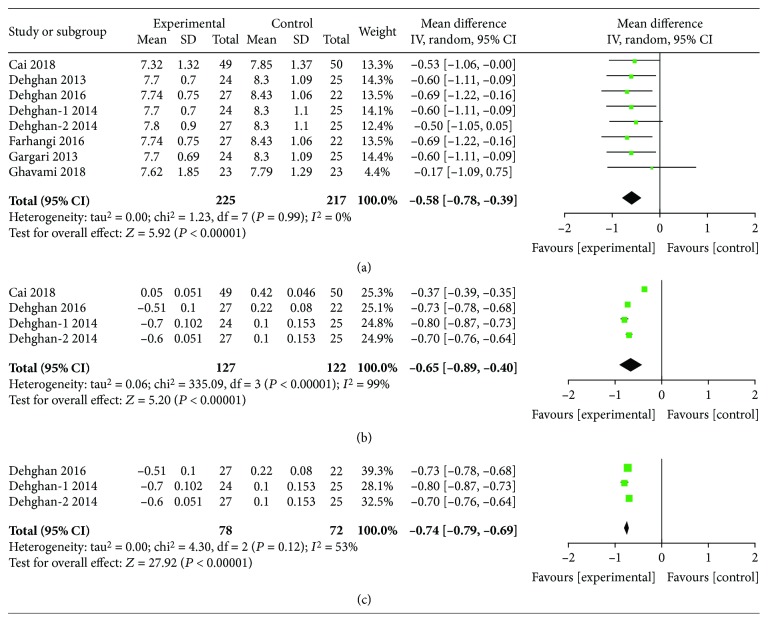
Findings of a meta-analysis of studies with continuation data on amelioration in HbA1c for ITC vs. control groups, with estimated MD and 95% CI. (a) Postintervention data of HbA1c, (b) reduction data of HbA1c, and (c) subgroup analysis for reduction data according to the baseline BMI level.

A total of 11 studies analyzed FPG for all subjects, and 8 articles studied FI and HbA1c. The FPG levels of all subjects were significantly lower in the ITC intervention group (*I*^2^ = 0%; *P* < 0.00001), and the reduction data of the FPG levels before and after the intervention were also more significant in the ITC group (MD, -16.42; 95% CI, -17.58 to -15.25; *I*^2^ = 41%; *P* < 0.00001) (Figures 4(a) and 4(b)). After ITC consumption, the FI level was lower than that in the control group, but there was significant heterogeneity (*I*^2^ = 74%; *P* = 0.02) (Figure 5(a)). Subgroup analysis based on the baseline BMI could eliminate the heterogeneity (*I*^2^ = 0%; *P* < 0.00001) (Figure 5(b)). The reduction data of FI after the ITC intervention were not significant compared with the control group (MD, -3.29; 95% CI, -6.88-0.3; *I*^2^ = 99%; *P* = 0.07), but subgroup analysis found that the ITC group has a significant FI reduction (*I*^2^ = 0%; *P* < 0.00001) (Figures 5(c) and 5(d)). The postintervention HbA1c level and change data of HbA1c consistently showed that the ITC group had absolute advantages, but the change data had heterogeneity (MD, -0.58%; 95% CI, -0.78% to -0.39%; *I*^2^ = 0%; *P* < 0.00001; and MD, -0.65%; 95% CI, -0.89% to -0.4%; *I*^2^ = 99%; *P* < 0.00001, respectively). Subgroup analysis based on the BMI could eliminate partial heterogeneity (*I*^2^ = 53%; *P* < 0.00001) (Figures 6(a)–6(c)).

### 3.4. Ameliorated Effect of ITC Intervention on IR

**Figure 7:**
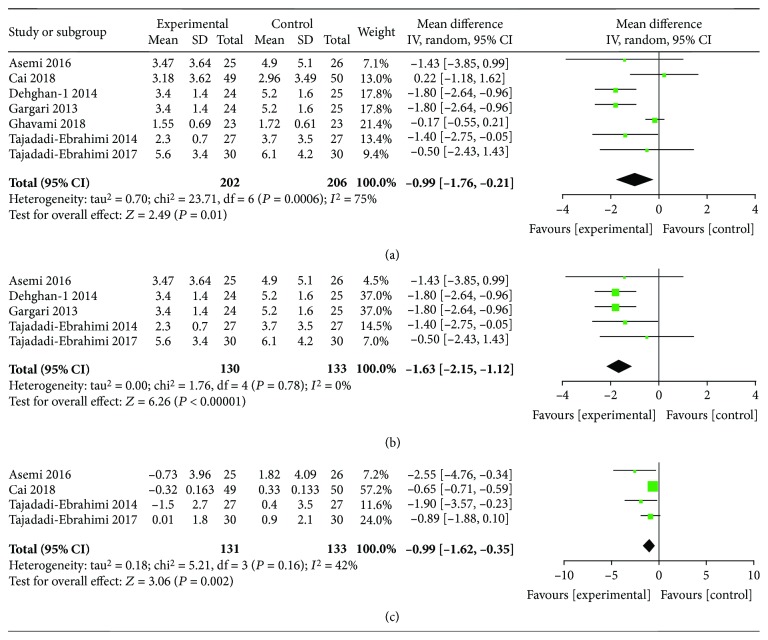
Findings of a meta-analysis of studies with continuation data on amelioration in HOMA-IR for ITC vs. control groups, with estimated MD and 95% CI. (a) Postintervention data of HOMA-IR, (b) subgroup analysis for postintervention data according to the baseline BMI level, and (c) reduction data of HOMA-IR.

**Figure 8:**
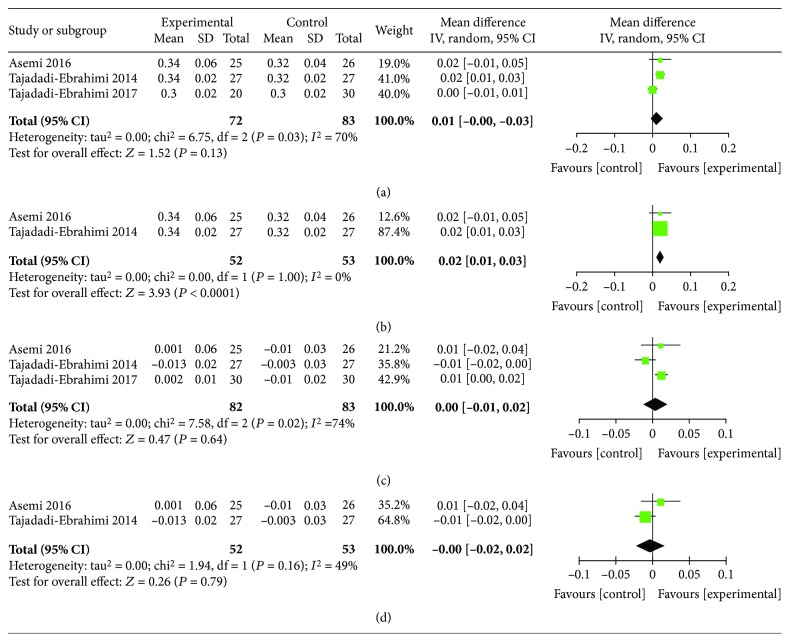
Findings of a meta-analysis of studies with continuation data on improvement in QUICKI for ITC vs. control groups, with estimated MD and 95% CI. (a) Postintervention data of QUICKI, (c) reduction data of QUICKI, and (b, d) subgroup analysis according to the baseline BMI level.

The fasting IR index mainly includes the HOMA-IR and the QUICKI. ITC intervention significantly ameliorated HOMA-IR, either the postintervention HOMA-IR level or the reduction data of HOMA-IR (MD, -0.99; 95% CI, -1.76 to -0.21, *I*^2^ = 75%, *P* = 0.01; and MD, -0.99; 95% CI, -1.62 to -0.35, *I*^2^ = 42%, *P* = 0.002, respectively). Heterogeneity can be eliminated by subgroup analysis according to the baseline BMI (*I*^2^ = 0%; *P* < 0.00001) (Figures 7(a)–7(c)). Only 3 articles studied QUICKI. Meta-analysis found that there was no statistical difference between the ITC and control groups on postintervention data of QUICKI (MD, 0.01; 95% CI, 0.00-0.03; *I*^2^ = 70%; *P* = 0.13). According to the dose of ITC intake, the subgroup analysis showed the statistically significant difference after the study of low-dose ITC intake was excluded (MD, 0.02; 95% CI, 0.01-0.03; *I*^2^ = 0%; *P* < 0.0001). However, there was no statistical difference in QUICKI reduction between the two groups (MD, 0.00; 95% CI, -0.01-0.02; *I*^2^ = 74%; *P* = 0.64) and so did subgroup analysis (*I*^2^ = 49%; *P* = 0.79) (Figures 8(a)–8(d)).

### 3.5. Systematic Review of ITC Intervention on the Glycometabolism and Homeostasis Model

Data from 14 other studies, including T2DM (5 studies) and simple obese (9 studies) patients, could not be pooled (Table 2). Except Alles et al.' study [36] which does not give the baseline BMI data, all subjects' average baseline BMI was greater than 28 kg/m^2^. In the ITC intervention group, the daily dose ranged from 4 to 30 g and the duration ranged from 2 weeks to 4 months. It was found that the conclusions about blood glucose, insulin, HbA1c, and HOMA-IR were complicated. Only Aliasgharzadeh et al. [19] found that ITC intervention could significantly decrease FPG and HbA1c in T2DM patients (*P* < 0.05); none of the other four studies reached a positive conclusion. Moreover, 9 studies on obese patients found that the results of blood glucose, insulin, and HbA1c were controversial. However, only Genta et al.'s [20] study on HOMA-IR concluded that ITC intervention was statistically significant, while the other six studies did not.

## 4. Discussion

IR is not only the central link and treatment target of T2DM but also one of the mechanisms of other diseases secondary to T2DM. Studies have found that IR and hyperglycemia can increase the risk of adverse cardiovascular events [44] and suggested a link between IR in T2DM patients and cognitive dysfunction [45] and Parkinson's disease [46]. The main index to evaluate IR is hyperinsulinemic-euglycemic clamp, HOMA-IR, and QUICKI. In recent years, many RCTs about the effect of ITC on the amelioration of blood glucose and IR have been reported [19, 20] and some systematic reviews on the effect of ITC on blood lipid, triacylglycerols, and chronic constipation have been carried out [10, 47, 48]. However, the meta-analysis of ITC-ameliorated IR has not been conducted. In the present study, we made this review involving 25 RCTs of parallel or crossover; to our knowledge, this was the first systematic analysis to evaluate the role of ITC supplementation in ameliorating IR in T2DM and obese patients.

We found the explicit effect of ITC supplementation on glycometabolism and HOMA-IR amelioration in T2DM with obesity. Postintervention and reduction outcomes of FPG, HbA1c, and HOMA-IR were significantly ameliorated after ITC supplementation in meta-analysis. Insulin secretion decreased significantly after inulin intake, but there was no significant difference between the reduction data of two groups (*I*^2^ = 99%, *P* = 0.07). With the heterogeneity, we noted that the baseline BMI may affect outcomes. In the study where the baseline BMI was greater than the 28 kg/m^2^, the subgroup analysis found that the difference was statistical and the heterogeneity was eliminated. The data of QUICKI, another index of IR, was collected in three studies and showed that there was no statistical difference between the two groups. Based on the characteristics of these studies, we speculated that the outcome may be related to the daily intakes of ITC. However, because the number of studies is too small, the conclusion cannot be generalized. As data could not be pooled, we systematically reviewed nine studies of obese people. The indicator of IR is the HOMA-IR; six studies concluded that ITC was ineffective.

As well known, obesity is closely associated with type 2 diabetes. However, this study found that ITC consumption had a controversial effect on IR in simple obesity but a significant result in T2DM, especially in T2DM with obesity. ITC could not be digested and absorbed in the small intestine but could be fermented by the microbial flora in the large bowel [49, 50]. In addition, ITC can modulate the composition of gut microbiota and increase the formation of short-chain fatty acids (SCFAs) in the process of intestinal fermentation [51, 52]. SCFAs had been shown to increase insulin sensitivity, improve glucose tolerance, and reduce *β*-cell apoptosis in obese and diabetic animals [53, 54] and could also stimulate intestinal gluconeogenesis [55]. Moreover, several mechanisms can explain the beneficial effects of a diet containing inulin on metabolism. It might be attributed to their impact on gene expression [56] and modulation of the intestinal microbiota, SCFAs, and hormone axis, especially with regard to increased promotion of the hormone glucagon-like peptide-1 [57]. In addition, changes in the levels of gut hormones like peptide YY [58] and activation of the lipopolysaccharide Toll-like receptor-2 were also mechanisms [59]. The effect of ITC on IR was related to the increase of specific intestinal flora [60]; maybe, ITC had different effects on the intestinal microbes in simple obesity and T2DM.

There were some limitations in this study. First, the amount of studies included in this meta-analysis was small and some studies have small sample size, so the random error existed and bias of results may occur. Second, the oral glucose tolerance test is recommended to assess IR in clinical practice commonly [61], but none of the studies included in this meta-analysis conducted OGTT tests, so we did not obtain 2 h postprandial blood glucose data. Third, subgroup analysis on the baseline BMI or inulin intake dosage in T2DM may have an unpredictable bias and the BMI was an independent factor required for Cox model analysis in multiple clinical trials. It suggested that much clinic trials will be needed to clarify the impact of ITC supplementation on the prevention and treatment of metabolic diseases. Finally, this meta-analysis did not have strict exclusion criteria in order to include all related studies as much as possible. We only excluded the studies that have normal people subjects. However, the studies included had some common exclusion criteria, such as subjects had a history of gastrointestinal, pancreatic, or cardiovascular disease, renal, thyroid, or liver disturbance, being pregnant or lactating, consuming pre- or probiotic products, antibiotics, antidiarrheal, anti-inflammatory, or laxative drugs, or if the subjects had a daily fiber intake > 30 g. In addition, the differences in these studies, such as baseline BMI levels in subjects, duration of diabetes, and dose and duration of ITC, were subgroup analyzed to determine the significance of these factors. Therefore, we think that the above limitations did not influence our conclusion that ITC supplementation can ameliorate IR in T2DM, especially in patients with obesity.

## 5. Conclusions

This meta-analysis indicates that the supplementation of ITC is efficacious in glycemic control and IR amelioration in T2DM, especially in obese T2DM patients. However, it is controversial in obese patients. Meanwhile, more randomized, double-blind, and large-sample-sized trials of ITC for T2DM and simple obese are needed in the future to validate or revise the result of this work.

## Figures and Tables

**Table 1:** The characteristics of the studies included in meta-analysis.

Study	Design	Sample size (ITC/Con)	Gender (M/W)	Age (years) ITC vs. Con	Baseline weight (kg) ITC vs. Con	Baseline BMI (kg/m^2^) ITC vs. Con	ITC dose (g/d)	Duration	ITC total dose (g)	Control	Diabetes duration (year) ITC vs. Con	Hypoglycemic agents
Ghavami 2018 [24]	R, DB, P	46 (23/23)	10/13 10/13	41.5 ± 6.27 42.73 ± 5.95	81.87 ± 11.46 79.91 ± 14.6	27.71 ± 4.628.79 ± 4.77	10	6 w	420	Starch powder	8.78 ± 4.67 9.86 ± 4.95	Glucose-lowering drugs
Gargari 2013 [25]	R, TB, P	49 (24/25)	0/24 0/25	47.77 ± 10.14 48.69 ± 9.74	75.4 ± 11.31 70.53 ± 11.05	31.61 ± 4.09 29.9 ± 4.24	10	2 m	600	Maltodextrin	7.33 ± 5.42 5.33 ± 4.6	Metformin Glibenclamide
Dehghan 2013 [23]	R, P	49 (24/25)	0/24 0/25	47.8 ± 10.1 48.7 ± 9.7	75.45 ± 11.3 70.5 ± 11.05	31.6 ± 4.09 29.9 ± 4.2	10	8 w	560	Maltodextrin	7.3 ± 5.4 5.3 ± 4.6	Metformin Glibenclamide
Dehghan 2016 [24]	R, DB, P	49 (27/22)	0/27 0/22	48.07 ± 8.7 48.61 ± 9.16	74.96 ± 10.33 71.43 ± 10.76	31.43 ± 3.5 29.98 ± 4.01	10	2 m	600	Maltodextrin	7.96 ± 5.15 5.5 ± 4.21	NG
Dehghan-1 2014 [25]	R, TB, P	49 (24/25)	0/24 0/25	47.8 ± 10.1 48.7 ± 9.7	75.4 ± 11.3 70.5 ± 11.05	31.6 ± 4.09 29.9 ± 4.2	10	8 w	560	Maltodextrin	7.3 ± 5.4 5.3 ± 4.6	Metformin Glibenclamide
Cai 2018 [26]	R, DB, P	99 (49/50)	16/33 22/28	60.94 ± 5.35 60.16 ± 5.84	71.82 ± 12.82 72.12 ± 12.40	27.86 ± 3.49 27.69 ± 3.79	7.5	12 w	630	Placebo milk powder	117.07 ± 206.6 73.80 ± 56.79 (m)	Oral diabetic medication
Dehghan-2 2014 [27]	R, TB, P	52 (27/25)	0/27 0/25	48.4 ± 8.4 48.7 ± 9.7	76.0 ± 12.2 70.5 ± 11.0	31.9 ± 4.5 29.9 ± 4.2	10	8 w	560	Maltodextrin	8.5 ± 5.0 5.3 ± 4.6	Metformin Glibenclamide
Farhangi 2016 [28]	R, DB, P	49 (27/22)	0/27 0/22	48.07 ± 8.7 48.61 ± 9.16	NG	31.43 ± 3.5 29.98 ± 4.01	10	2 m	600	Maltodextrin	7.96 ± 5.15 5.5 ± 4.21	Metformin Glibenclamide
Asemi 2016 [29]	R, DB, C	51 (25/26)	16/35	52.9 ± 8.1	77.59 ± 13.65 78.28 ± 13.42	29.88 ± 4.77 30.15 ± 5.07	2.7	6 w	113.4	Control food	NG	NG
Tajadadi-Ebrahimi 2014 [30]	R, DB, P	81 (27/27)	5/22 5/22	51.3 ± 10.4 53.4 ± 7.5	80.6 ± 15.2 76.8 ± 12.1	30.8 ± 5.9 30.5 ± 4.1	8.4	8 w	470.4	Control bread	NG	Metformin Glibenclamide
Tajadadi-Ebrahimi 2017 [31]	R, DB, P	60 (30/30)	NG	64.2 ± 12 64 ± 11.7	79.2 ± 15.4 74.3 ± 13.7	32.3 ± 6.0 29.6 ± 4.6	0.8	12 w	67.2	Placebo	NG	NG

Abbreviations: R: randomized; DB: double-blind; TB: triple-blind; C: crossover; P: parallel; ITC: inulin-type carbohydrate; Con: control; BMI: body mass index; w: weeks; m: months; NG: not given.

**Table 2:** The characteristics of the studies included in a systematic review.

Study	Subjects	Sample size (ITC/Con)	Baseline weight (kg) ITC vs. Con	Baseline BMI (kg/m^2^) ITC vs. Con	ITC dose (g/d)	Duration	Control	Diabetes duration (year) ITC vs. Con	Hypoglycemic agents	Positive indices	Negative indices
Aliasgharzadeh 2015 [16]	T2DM	52 (27/25)	76.0 ± 12.2 70.5 ± 11.0	31.9 ± 4.0 29.9 ± 4.1	10	8 w	Maltodextrin	8.50 (5.00) 5.30 (4.60)	Metformin Glibenclamide	FPG and HbA1c (*P* < 0.05)	—
Roshanravan 2017 [18]	T2DM	59 (15/15)	86.07 ± 10.33 81.74 ± 16.64	30.37 ± 2.82 30.86 ± 5.41	10	45 d	Starch powder	1.61 ± 0.34 1.43 ± 0.31	First-line preventive medications	—	FPG, insulin, HbA1c, and HOMA-IR
Bonsu 2012 [32]	T2DM	26 (12/14)	85.6 ± 17.9 83.0 ± 14.1	31.0 ± 4.5 29.7 ± 4.3	10	12 w	Xylitol	6.0 ± 3.7 6.0 ± 4.4	NG	—	FPG and HbA1c
Alles 1999 [33]	T2DM	20 (20/20)	NG	NG	15	20 d	Glucose	NG	Glucose-lowering medication	—	FPG
Luo 2000 [19]	T2DM	10 (10/10)	73.6 ± 3.5	28.0 ± 1.0	20	4 w	Sucrose	11 ± 2	Metformin Sulfonylurea	—	Basal hepatic glucose, FPG, insulin, and HbA1c
Guess 2015 [20]	Obesity	39 (20/19)	88.2 ± 14.0 83.4 ± 19.7	30.8 ± 4.1 30.0 ± 2.3	30	18 w	Cellulose	None	None	FPG (*P* = 0.005)	Insulin and HOMA-IR
Guess 2016 [34]	Obesity	40 (20/20)	96.5 ± 5.4 88.3 ± 3.7	31.1 ± 1.0 28.4 ± 2.2	30	2 w	Cellulose	None	None	iAUC_(0-30 min)_ insulin (*P* < 0.04) and iAUC_(0-60 min)_ insulin (*P* < 0.04)	FI, tAUC glucose/insulin, and HOMA-IR
Rebello 2015 [35]	Obesity	28 (14/14)	95 ± 16.1 90.9 ± 19.5	34.7 ± 5.8 31.5 ± 5.1	4	4 w	Placebo	None	None	Blood glucose tolerance (*P* = 0.008)	Insulin sensitivity, HOMA-IR, and HbA1c
Dewulf 2013 [36]	Obesity	30 (15/15)	99.1 ± 16.3 97.5 ± 15.8	36.1 ± 4.1 36.1 ± 4.1	16	3 m	Maltodextrin	None	None	Post-OGTT glycemia (*P* < 0.05)	HbA1c, FPG, insulin, post-OGTT insulinemia, and HOMA index
Daud 2014 [37]	Obesity	22 (12/10)	83.7 ± 4.9 83.7 ± 4.9	29.7 ± 1.0 31.1 ± 1.1	30	6 w	Cellulose	None	None	—	Glucose, insulin, HOMA-IR, and HOMA % *β*
de Luis 2013 [38]	Obesity	36 (18/18)	92.3 ± 11.3 106.4 ± 16.2	35.9 ± 3.4 39.2 ± 7.2	9.84	1 m	Control cookie	None	None	—	FPG, insulin, and HOMA-IR
Parnell 2009 [39]	Obesity	39 (21/18)	83.4 ± 13.0 80.2 ± 12.8	30.4 ± 3.4 29.8 ± 4.0	21	12 w	Maltodextrin	None	None	Postprandial insulin (*P* < 0.05)	Postprandial glucose and FPG
Genta 2009 [17]	Obesity	35 (20/15)	89.2 ± 11.4 90.7 ± 10.3	34.0 ± 2.0 33.0 ± 3.0	10	4 m	Control syrup	None	None	FI and HOMA-IR (*P* < 0.05)	FPG
Tovar 2012 [40]	Obesity	59 (30/29)	76.55 ± 10.96 76.45 ± 11.07	30.74 ± 3.87 30.86 ± 4.47	10	3 m	No treatment	None	None	—	FPG

Abbreviations: T2DM: type 2 diabetes mellitus; R: randomized; DB: double-blind; TB: triple-blind; ITC: inulin-type carbohydrate; Con: control; BMI: body mass index; FPG: fasting plasma glucose; FI: fasting insulin; AUC: area under the curve; OGTT: oral glucose tolerance test; HOMA-IR: homeostasis model assessment insulin resistance.
